# *Morganella morganii* Pericarditis in a Patient with Multiple Myeloma

**DOI:** 10.1155/2013/452730

**Published:** 2013-09-11

**Authors:** Takafumi Nakao, Masahiro Yoshida, Hiroshi Kanashima, Takahisa Yamane

**Affiliations:** Department of Hematology, Osaka City General Hospital, 2-13-22 Miyakojima-hondori, Miyakojima-ku, Osaka 534-0021, Japan

## Abstract

Purulent pericarditis caused by *Morganella morganii* is extremely rare. We report herein a case of a 61-year-old man who presented with chest pain and dyspnea fourteen days after chemotherapy for multiple myeloma. Echocardiogram and computed tomography revealed a massive pericardial effusion and associated cardiac tamponade. Pericardiocentesis was performed. Pericardial fluid was found to be purulent, and *Morganella morganii* was isolated from the fluid. The patient was successfully treated with antibiotic therapy and surgical drainage of the fluid. *Morganella morganii* should be considered a possible pathogen when immunocompromised patients develop purulent pericarditis.

## 1. Introduction

*Morganella morganii* is a gram-negative facultative anaerobe that is ubiquitous in the environment. The organism may cause many kinds of opportunistic infection; however, it is very rare for it to be a cause of purulent pericarditis. To date, only four articles in English [1–4] and one in Russian [5] have been published on purulent pericarditis caused by *Morganella morganii*.

Multiple myeloma is a neoplastic plasma cell dyscrasia characterized by a serum monoclonal protein, anemia, skeletal destruction, renal insufficiency, and hypercalcemia. Patients with multiple myeloma are at high risk of bacterial infections. Here, we describe a rare case of purulent pericarditis caused by *Morganella morganii* in a patient with multiple myeloma. Pericarditis was successfully managed with antibiotic therapy and pericardial drainage. 

## 2. Case Report 

**Figure 1:**
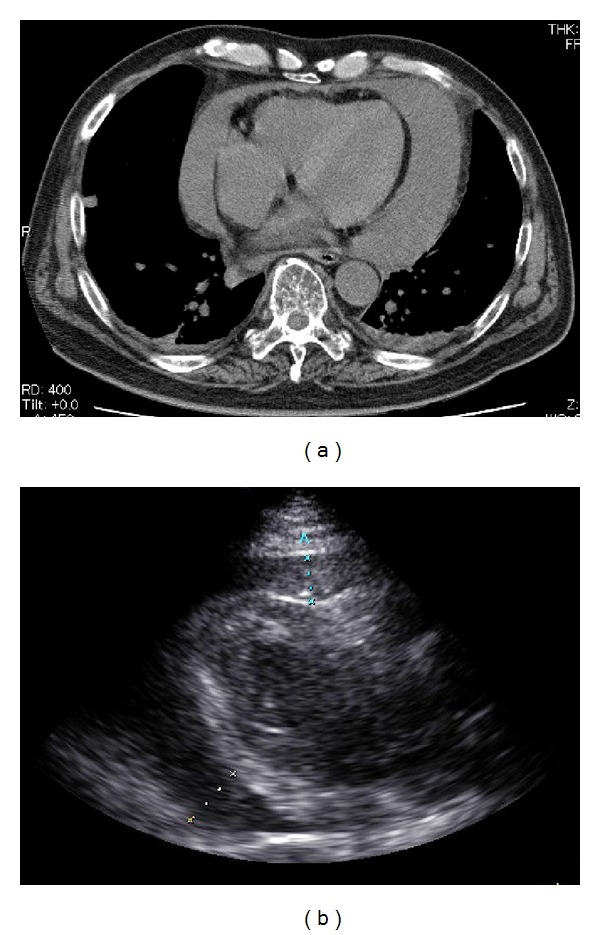
Chest CT scan showing large pericardial effusion (a) and echocardiogram showing circumferential pericardial effusion (b).

A 61-year-old male was diagnosed with multiple myeloma (immunoglobulin IgG-*κ* type) in 2006. He underwent 2 cycles of combination chemotherapy consisting of vincristine, doxorubicin, and dexamethasone (VAD) that were very effective. Subsequently, he underwent high-dose chemotherapy (melphalan: 200 mg/m^2^) followed by tandem autologous peripheral blood stem cell transplantation. Although he achieved complete remission, multiple new lytic bone lesions were found in 2010. Salvage treatments were initiated, including MP therapy (melphalan and predonisolone) or bortezomib which were associated with a temporary response. In February 2011, lenalidomide was started in combination with dexamethasone. After 2 cycles of treatment, the patient developed massive pulmonary embolism. He underwent inferior vena cava filter placement, and thrombus suction was performed. In August 2011, he received repeat VAD therapy. Fourteen days after chemotherapy, when he was neutropenic, he had frequent bouts of diarrhea, high-grade fever, and dyspnea. One set of peripheral blood culture yielded a gram-negative rod within 24 h of incubation. The isolate was subsequently identified as *Morganella morganii*. Echocardiogram and computerized tomography (CT) revealed a massive pericardial effusion (Figure 1) and associated cardiac tamponade. Pericardiocentesis and pericardial fluid drainage was performed, and 1,200 mL of heavily blood-stained fluid was drained. The results of the pericardial fluid analysis were as follows: red blood cell count, 1.6 million cells/mm^3^; white blood cell count, 185,600 cells/mm^3^ with neutrophilia of 95.5%; total protein, 7.1 g/dL; glucose < 5 mg/dL; lactate dehydrogenase, 4,390 U/L. *Morganella morganii* grew in cultures of the pericardial fluid, and no myeloma cells were detected in the fluid. The patient was started on intravenous cefozopran. The chest drain was removed after 5 days and pericarditis responded well to 5-week antibiotic therapy. After pericardial fluid drainage and antibiotic treatment, there was no additional pericardial fluid accumulation, and follow-up blood cultures were negative. The patient had an uneventful recovery and did not experience any recurrence of pericarditis 2 years after his illness. 

## 3. Discussion

*Morganella morganii* is a gram-negative facultative anaerobe that is commonly found in the environment and in the intestinal tracts of humans as normal flora. Despite its widespread distribution, it is a relatively uncommon cause of clinical human infections [6]. Urinary tract infection is probably the most common infection caused by *Morganella morganii* in humans [7]. *Morganella morganii* has also been documented as causing skin and soft tissue infections [8], peritonitis [9], meningitis [10], and bacteremia/sepsis [11]. However, *Morganella morganii* pericarditis is extremely rare. Common bacterial pathogens associated with purulent pericarditis include *Streptococcus pneumoniae* most frequently, *Staphylococcus aureus*, and gram-negative organisms such as *Proteus* species, *Escherichia coli*, *Pseudomonas* species, and *Klebsiella* species. Only four articles have been published in English on purulent pericarditis caused by *Morganella morganii* [1–4]. In three of these four cases, the patients were immunocompromised due to underlying diseases. One was a patient with X-linked agammaglobulinemia [3]. The other two patients developed pericarditis after allogeneic bone marrow transplantation for hematological malignancies. In these two patients, splenectomies were performed before the onset of pericarditis [1, 2]. Our patient also had a number of features predisposing to infection including multiple myeloma, immunosuppressive therapy, and pulmonary embolism treated with inferior vena cava filter placement. Our experience suggests that *Morganella morganii* should be considered a possible pathogen when immunocompromised patients develop purulent pericarditis.

Pericardial involvement and cardiac tamponade are rare complications of multiple myeloma. Cardiac tamponade has been reported to be caused by amyloidosis, infections, or plasma cell infiltration in patients with multiple myeloma. In our case, the patient was diagnosed with infectious pericarditis because the pericardial fluid was purulent and *Morganella morganii* was isolated from the fluid. No myeloma cells were detected in the fluid. We suspect that the patient developed pericardial infection as a result of hematogenous spread because *Morganella morganii* was isolated from both pericardial and blood cultures. Moreover, we suspect that the primary focus of the infection was the intestines because *Morganella morganii* is part of the normal flora of the intestine, and the patient had early gastrointestinal symptoms including several bouts of diarrhea prior to the onset of the pericarditis. The prognosis of purulent pericarditis remains poor, primarily due to the lack of prompt recognition and initiation of treatment. Intravenous antimicrobial therapy should be started as soon as the diagnosis of purulent pericarditis is suspected, and surgical drainage should always be considered to relieve cardiac tamponade. 

In conclusion, we report a very rare case of purulent pericarditis with cardiac tamponade caused by *Morganella morganii*. Prompt institution of antibiotic therapy and pericardial drainage resulted in a favorable outcome for our patient.
